# Supplementary material for: Chagas Disease Diagnosis with Trypanosoma cruzi-Exclusive Epitopes in GFP
Source: Vaccines (Basel). 2024 Sep 8;12(9):1029. doi: 10.3390/vaccines12091029 (PMC11435546; doi:10.3390/vaccines12091029)
Supplement: Supplementary file 1 [file vaccines-12-01029-s001.zip › vaccines-3045530-supplementary.pdf]

Supplemental Data

Table S1: Insertion size and position of extraneous sequences into fluorescent proteins.

Insertion size	Position	Reference
25-50 aa	ß1-ß2 (24)	1
25-50 aa	ß3-ß4 (50)	1
13 aa	ß4-ß5 (102)	2
6-20 aa	ß5-ß6 (116)	3,4
≤18 aa	ß7-ß8 (157)	3
≤20 aa	ß8-ß9 (172)	2-4
25-50 aa	ß8-ß9 (173)	1
≤20 aa	ß9-ß10 (189)	3,4

1. Kiss, C. et al. Antibody binding loop insertions as diversity elements. *Nucleic Acids Res* **34**, e132 (2006).
2. Pavoor, T. V., Cho, Y. K. & Shusta, E. V. Development of GFP-based biosensors possessing the binding properties of antibodies. *Proc Natl Acad Sci U S A* **106**, 11895-11900 (2009).
3. Zhong, J. Q., Freyzon, Y., Ehrlich, D. J. & Matsudaira, P. Enhanced detection sensitivity using a novel solid-phase incorporated affinity fluorescent protein biosensor. *Biomol Eng* **21**, 67-72 (2004).
4. Abedi, M. R., Caponigro, G. & Kamb, A. Green fluorescent protein as a scaffold for intracellular presentation of peptides. *Nucleic Acids Res* **26**, 623-630 (1998).

Table S2: Amino acids in the ß-barrel altered by directed evolution to rapidly folding and highly stable proteins.

**Fluorescent Protein**	**ß1**	**ß2**		**ß3**	**ß4**	**ß5**		**ß6**		**ß7**		**ß8**				**ß10**			**ß11**
	**16**	**28**	**30**	**43**	**99**	**105**	**111**	**124**	**128**	**149**	**153**	**163**	**166**	**167**	**171**	**205**	**206**	**208**	**223**
eGFP [1]	V	S	S	T	F	N	E	E	I	N	M	V	K	I	I	S	A	S	F
cycle-3 [2]	V	S	S	T	S	N	E	E	I	N	T	A	K	I	I	S	A	S	F
Split [3]	V	S	R	T	S	K	V	E	T	N	T	A	T	V	V	T	V	S	F
SuperFolder [4]	V	S	R	T	S	T	E	E	I	N	T	A	K	I	V	S	V	S	F
Superfast [5]	V	S	S	T	F	Y	E	V	I	N	M	V	K	I	I	S	A	S	F
Tri Split [3]	I	F	R	S	Y	T	E	E	I	K	T	A	T	I	V	T	I	L	Y
ß-receptacle	I	F	S	S	Y	T	E	V	I	N	T	A	T	V	V	T	I	L	Y

1. Cormack, B. P., Valdivia, R. H. & Falkow, S. FACS-optimized mutants of the green fluorescent protein (GFP). *Gene* **173**, 33-38 (1996).
2. Crameri, A., Whitehorn, E. A., Tate, E. & Stemmer, W. P. Improved green fluorescent protein by molecular evolution using DNA shuffling. *Nat Biotechnol* **14**, 315-319 (1996).
3. Cabantous, S. et al. A new protein-protein interaction sensor based on tripartite split-GFP association. *Sci Rep* **3**, 2854 (2013).
4. Pedelacq, J. D., Cabantous, S., Tran, T., Terwilliger, T. C. & Waldo, G. S. Engineering and characterization of a superfolder green fluorescent protein. *Nat Biotechnol* **24**, 79-88 (2006).
5. Fisher, A. C. & DeLisa, M. P. Laboratory evolution of fast-folding green fluorescent protein using secretory pathway quality control. *PLoS One* **3**, e2351 (2008).

Table S3: Peptides and their positions in the Spot Synthesis Analysis with epitopes in bold.

Spot	Peptide	Spot	Peptide	Spot	Peptide	Spot	Peptide
A1	MGSSHHHHHHSSGL	B11	KEDGNIL**KQKAAEA**	C21	-	E7	**GQAAAADK**GRDTLV
A2	HHHHSSGLVPRGSH	B12	L**KQKAAEATK**LKGH	C22	-	E8	**DK**GTDTLVNRIVLK
A3	GLVPRGSH**KFAELL**	B13	EATKLKGHKLEYNF	C23	-	E9	LVNRIVLKGIDFKE
A4	SH**KFAELLEQQKNA**	B14	GHKLEYNFNSHNVY	C24	-	E10	LKGIDFKEDGNIL**K**
A5	**LLEQQKNAQFP**GKA	B15	NFNSHNVYITADAE	D1	MGSSHHHHHHSSGL	E11	KEDGNIL**KQKAAEA**
A6	NAQFPGKASKGEEL	B16	VYITAD**AEPKPAEP**	D2	HHHHSSGLVPRGSH	E12	L**KQKAAEATK**LKGH
A7	KASKGEELFTGVVP	B17	**AEPKPAEPKS**TADK	D3	GLVPRGSH**KFAELL**	E13	EATKLKGHKLEYNF
A8	ELFTGVVPILIELD	B18	**EPKS**TADKQKNGIK	D4	SH**KFAELLEQQKNA**	E14	GHKLEYNFNSHNVY
A9	VPILIELDGDVNGH	B19	DKQKNGIKANFTVR	D5	**LLEQQKNAQFP**GKA	E15	NFNSHNVYITADAE
A10	LDGDVNGHKFFVRG	B20	IKANFTVRHNVEDG	D6	NAQFPGKASKGEEL	E16	VYITAD**AEPKPAEP**
A11	GHKFFVRGEGEGDA	B21	VRHNVEDGS**AEPKS**	D7	KASKGEELFTGVVP	E17	**AEPKPAEPKS**TADK
A12	RGEGEGDATIGKLS	B22	DGS**AEPKSAEPKP**G	D8	ELFTGVVPILIELD	E18	**EPKS**TADKQKNGIK
A13	DATIGKLSLKFICT	B23	**KSAEPKP**GSVQLAD	D9	VPILIELDGDVNGH	E19	DKQKNGIKANFTVR
A14	LSLKFICTTGKLKA	B24	**P**GSVQLADHTQQNT	D10	LDGDVNGHKFFVRG	E20	IKANFTVRHNVEDG
A15	CTTGKL**KAAAAPAK**	C1	ADHYQQNTPIGDGP	D11	GHKFFVRGEGEGDA	E21	VRHNVEDGS**AEPKS**
A16	**KAAAAPA**KLPVPWP	C2	NTPIGDGP**GTSEEG**	D12	RGEGEGDATIGKLS	E22	DGS**AEPKSAEPKP**G
A17	**A**KLPVPWPTLVTTL	C3	GP**GTSEEGSRGGSS**	D13	DATIGKLSLKFICT	E23	**KSAEPKP**GSVQLAD
A18	WPTLVTTLTYGVQC	C4	**EGSRGGSSMPS**DGP	D14	LSLKFICTTGKLKA	E24	**P**GSVQLADHTQQNT
A19	TLTYGVQCFSRYPD	C5	**SSMPS**DGPVLLPDN	D15	CTTGKL**KAAAAPAK**	F1	ADHYQQNTPIGDGP
A20	GCFSRYPDHMKRHD	C6	GPVLLPDNHYLSTQ	D16	**KAAAAPA**KLPVPWP	F2	NTPIGDGP**GTSEEG**
A21	PDHMKRHDFFKSAM	C7	DNHYLSTQTILLKD	D17	**A**KLPVPWPTLVTTL	F3	GP**GTSEEGSRGGSS**
A22	HDFFKSAMPEGYVQ	C8	TQTILLKDPNEL**SP**	D18	WPTLVTTLTYGVQC	F4	**EGSRGGSSMPS**DGP
A23	AMPEGYVQERTIYF	C9	KDPNEL**SPFGQAAA**	D19	TLTYGVQCFSRYPD	F5	**SSMPS**DGPVLLPDN
A24	VQERTIYFKDVKAA	C10	**SPFGQAAAGDK**ELK	D20	GCFSRYPDHMKRHD	F6	GPVLLPDNHYLSTQ
B1	YFKDV**KAAIAPA**DV	C11	**AAGDK**ELKRDHMVL	D21	PDHMKRHDFFKSAM	F7	DNHYLSTQTILLKD
B2	**AAIAPA**DVDGTYKT	C12	LKRDHMVLLEYVTA	D22	HDFFKSAMPEGYVQ	F8	TQTILLKDPNEL**SP**
B3	DVDGRYKTRAEVKF	C13	VLLEYVTAAGITLG	D23	AMPEGYVQERTIYF	F9	KDPNEL**SPFGQAAA**
B4	KTRAEVKFEGTGDK	C14	TAAGITLGMDELYK	D24	VQERTIYFKDVKAA	F10	**SPFGQAAAGDK**ELK
B5	KFEGF**GDKPDPFGQ**	C15	LGMDELYKEF**KQRA**	E1	YFKDV**KAAIAPA**DV	F11	**AAGDK**ELKRDHMVL
B6	**DKPSPFGQAAAADK**	C16	LYKEF**KQRAAEATK**	E2	**AAIAPA**DVDGTYKT	F12	LKRDHMVLLEYVTA
B7	**GQAAAADK**GRDTLV	C17	-	E3	DVDGRYKTRAEVKF	F13	VLLEYVTAAGITLG
B8	**DK**GTDTLVNRIVLK	C18	-	E4	KTRAEVKFEGTGDK	F14	TAAGITLGMDELYK
B9	LVNRIVLKGIDFKE	C19	-	E5	KFEGF**GDKPDPFGQ**	F15	LGMDELYKEF**KQRA**

B10	LKGIDFKEDGNIL**K**	C20	-	E6	**DKPSPFGQAAAADK**	F16	LYKEF**KQRAAEATK**

Table S4: BLAST analysis of epitope sequences.

Epitope Sequence (#)	% Identity	Organism
KFAELLEQQKNAQFPGK (1)	100	*T. cruzi*
		T. rangeli
		T. brucei gambiensi
	94.12	*L. braziliensis*
		T. rangeli
		Crithidia sp
KAAAAPA (2)	--	No identity
KAAIAPA (3)	--	No identity
GDKPSPFGQAAAADK (4)	100	*T.cruzi*
		Exophiala dermatitidis
		Aureobasidium melanogenum
	80	*Trypanosoma rangeli*
KQKAAEATK (5)	100	*T. cruzi*
		Ruminococcus sp.
AEPKPAEPKS (6)	100	*T.cruzi*
		Aspergillus melleus
AEPKSAEPKP (7)	100	*T.cruzi*
GTSEEGSRGGSSMPS (8)	100	*T.cruzi*
SPFGQAAAGDK (9)	100	*T.cruzi*
		T. brucei gambiensi
		T. brucei brucei
		T. brucei equiperdum
	90	*Trypanosoma conorhini*
		Trypanosoma rangeli
	83.33	*Streptococcus sp.*
KQRAAEATK (10)	100	*T.cruzi*
		Prevotella sp.
	88.89	*Eubacterium sp.*
		Alistipes sp.
DSSAHSTPSTPA (11)	100	*T.cruzi*
	90.91	*Pantoea sp.*
PPSGTENNKPATG (12)	100	*T.cruzi*
FGQAAAGDKPS (13)	100	*T.cruzi*

Figure S1

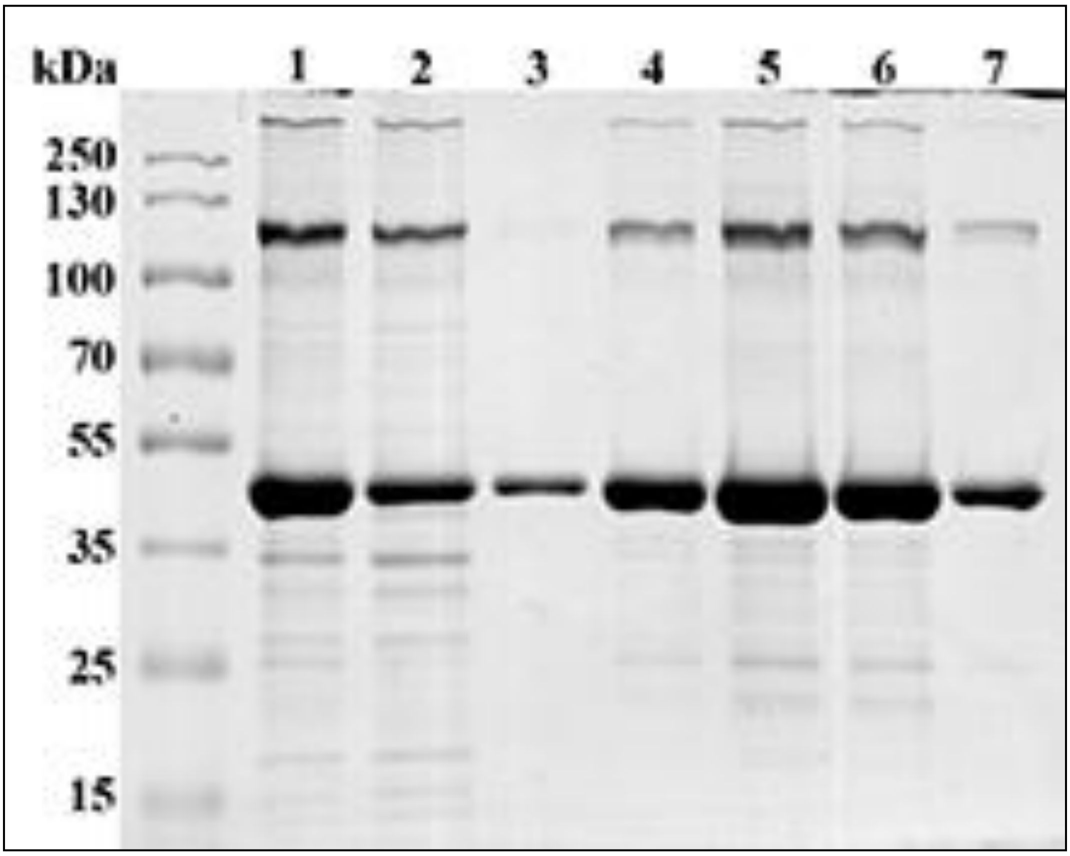

Figure S1: Representative purification of DxCruziV1. Recombinant protein was expressed in E. coli from a pET28a vector for 3hr with induction with IPTG. After the collection of bacteria from the culture, cells were lysed by sonification, and the inclusion bodies isolated by centrifugation. After three washes in the presence of detergent, the protein in the inclusion bodies was solubilized in 6M urea and applied to a Ni-column attached to an Aktä chromatography system. After loading and washing with 25 mM imidazole, bound protein was eluted by a gradient of imidazole to 500 mM. Equal volume samples of protein were separated on a 10% SDS-PAGE gel. First lane, standards for protein size; Lane 1, solubilized inclusion bodies (load); Lane 2, flow through; Lanes 3-7, consecutive fractions of protein eluted by the imidazole gradient.

Figure S2

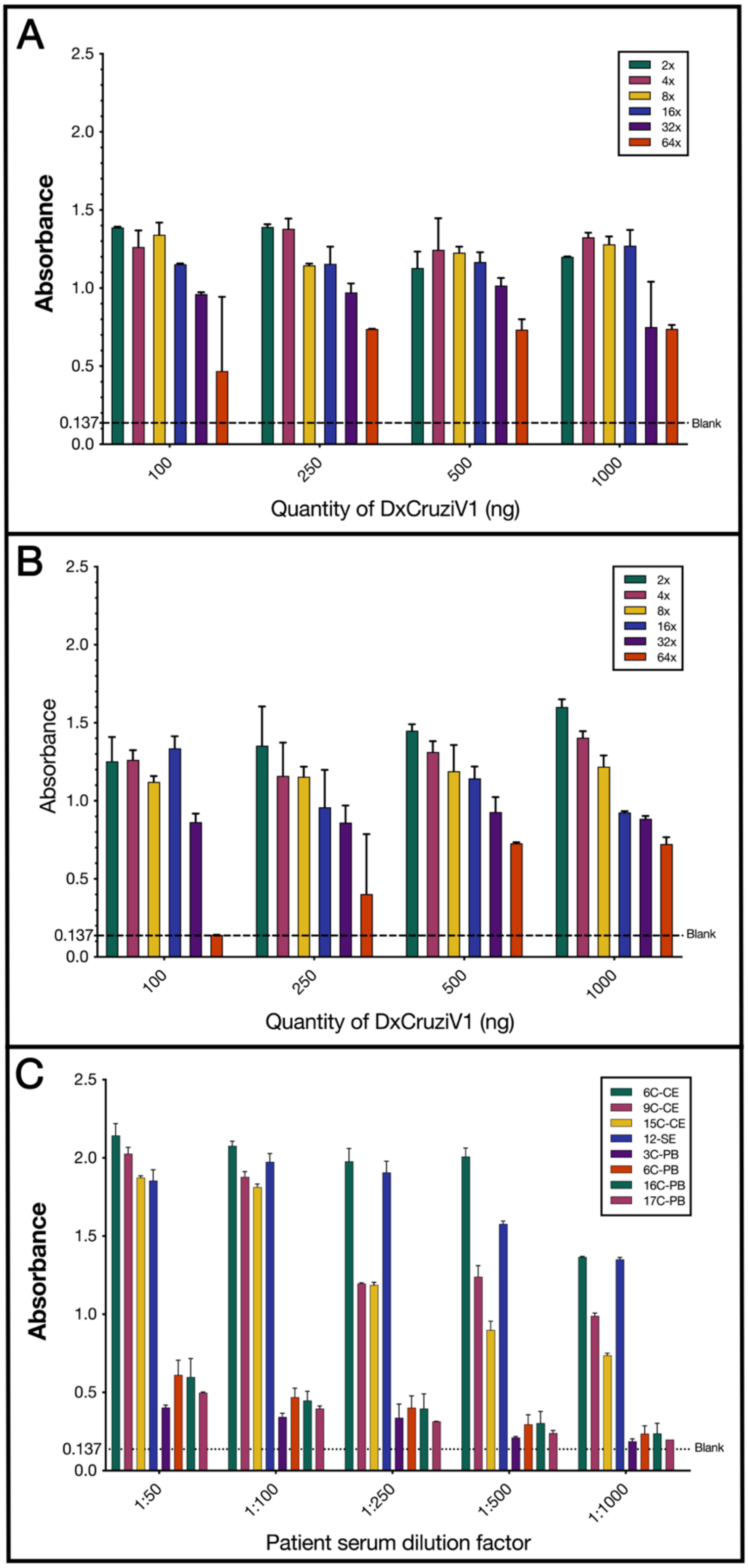

Figure S2: Optimization of ELISA conditions for sensibilization and patient serum dilution. The median signal and standard deviation measured from the application of WHO Chagas disease international standards 09/186 (Panel A) and 09/188 (Panel B) to

ELISA plates sensitized with different quantities of DxCruziV1. As the maximal signal at the highest dilution factor (1/64) was obtained with 500 ng, the same quantity was used to evaluate an optimal dilution factor for patient serum using two groups of four patients with either a high titer or a low titer (Panel C).

Figure S3

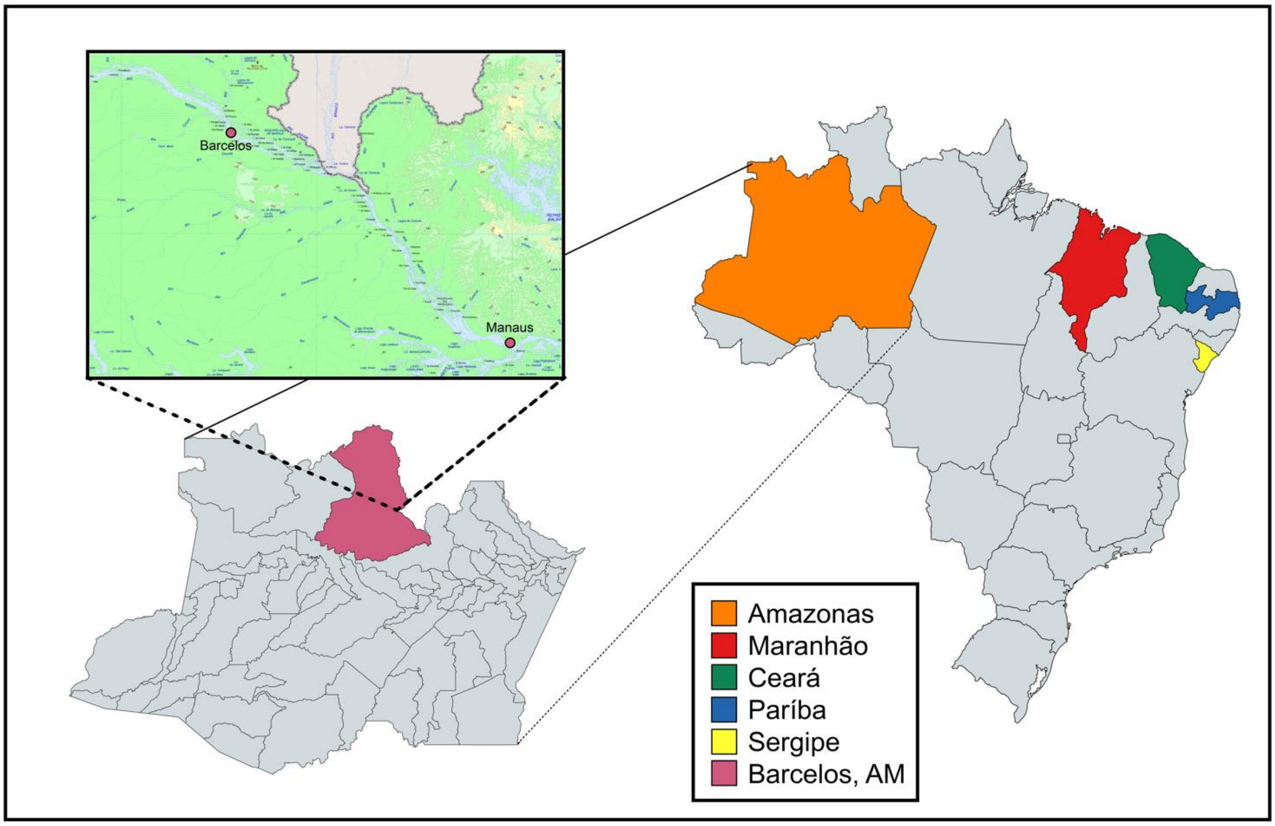

Figure S3: Geographical origins of patient serum in Brazil. Maps highlighting the regions where biological samples were collected. The full map of Brazil displays the states of Amazonas (orange); Maranhão (red); Ceará (green); Paraíba (blue) and Sergipe (yellow). The map of the state of Amazonas shows the region of Barcelos (mauve). The inset highlights the location of the Barcelos municipality on the banks of the Rio Negro, which is a multiday trip by boat from Manaus, AM, Brazil. Sources: Maps of Brazil and the state of Amazonas were generated on MapChart.net and region of Amazonas was modified from IBGE, 2015. (https://geoftp.ibge.gov.br/cartas_e_mapas/mapas_estaduais_e_distrito_federal/fisico/am_fisico1800k_2010.pdf).

Figure S4

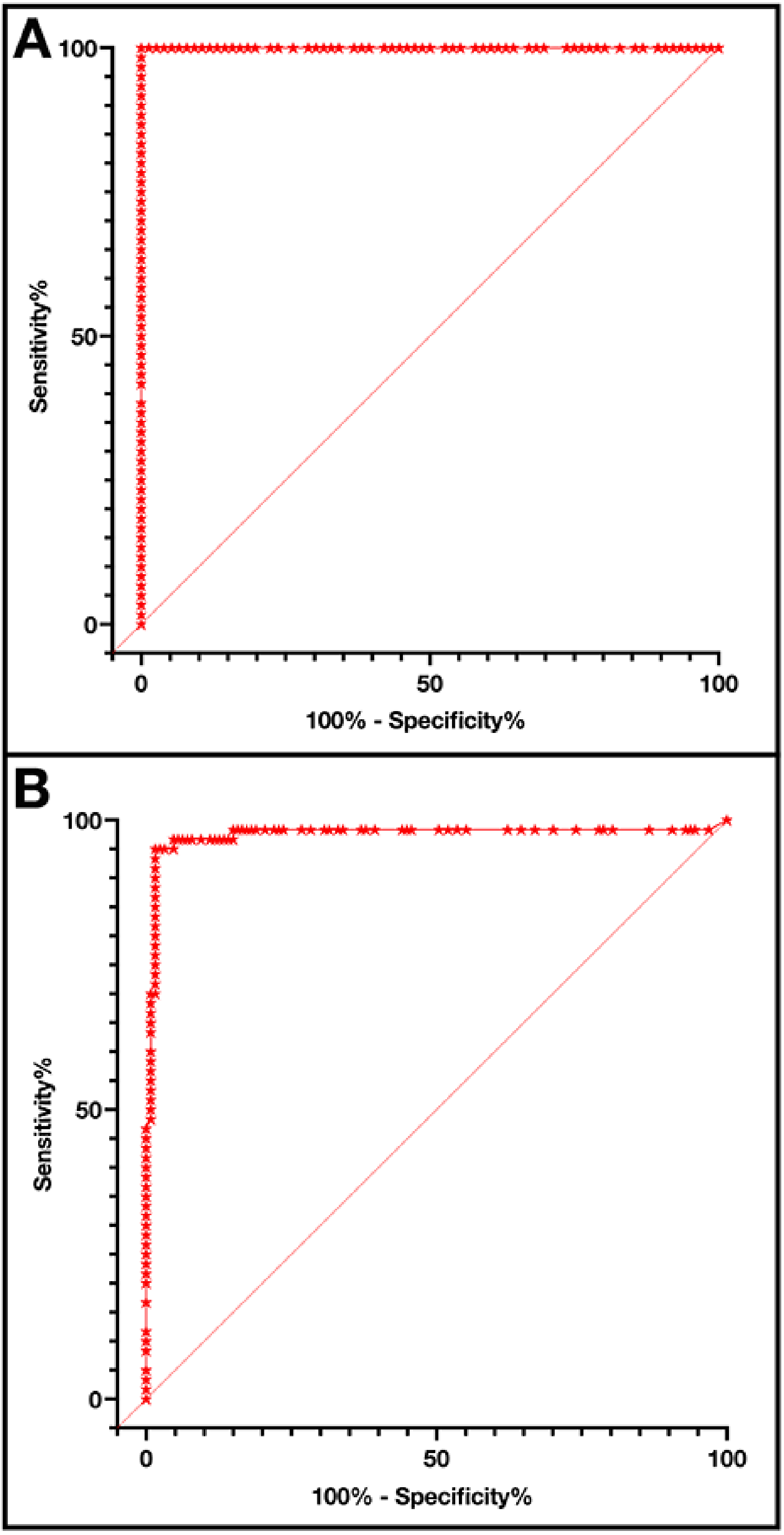

Figure S4: Receiver operating characteristic (ROC) of DxCruziV1 & DxCruziV2. The ROC analysis performed in

Prism10 (GraphPad) is plotted for DxCruziV1 (Panel A) and DxCruziV2 (Panel B).

Figure S5

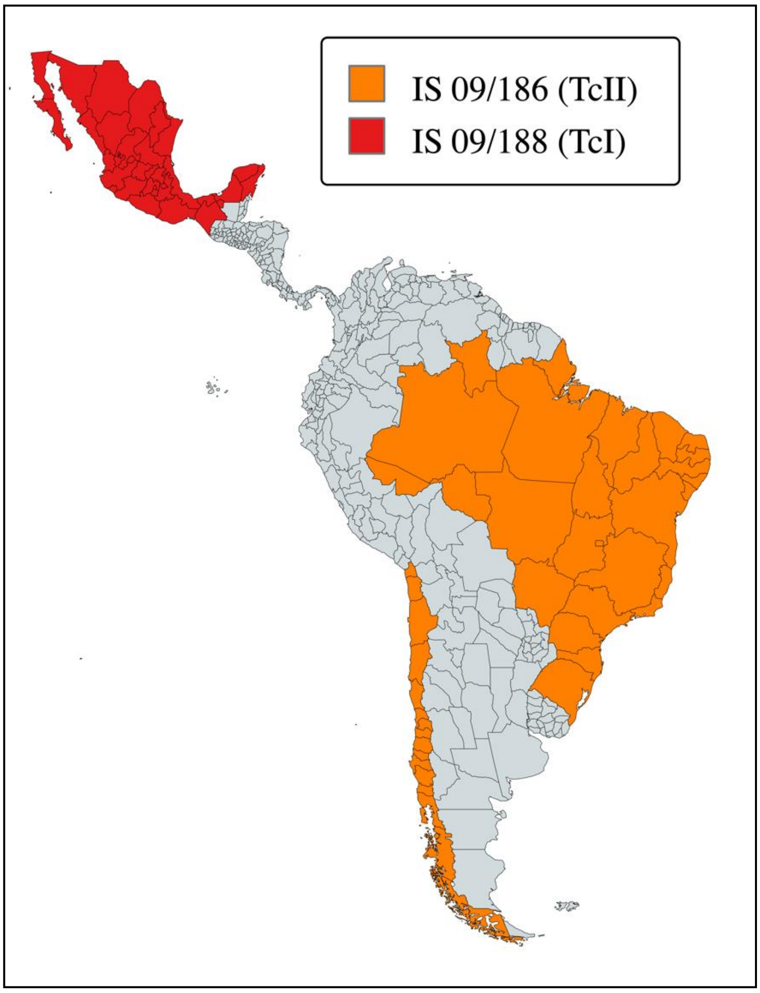

Figure S5: Geography of sample collection for WHO Biological Standards for Chagas disease. Map of Central and South America highlighting Mexico, Chile, and Brazil in orange where patient samples were collected to generate the International Biological Reference Standards. IS 09/188 (TcI) was from Mexico and IS 09/186 (TcII) from Chile and Brazil.
